# Chronic presentation of Boerhaave's syndrome

**DOI:** 10.1186/1471-230X-10-29

**Published:** 2010-03-12

**Authors:** Umer F Malik, Ryan Young, Hein D Pham, Alisha McCon, Brenda Shen, Richard Landres, Ahmed Mahmoud

**Affiliations:** 1 Department of Internal Medicine, San Joaquin County Hospital, 500 West hospital road, French Camp, California, 95231, USA; 2 Department of Anesthesiology, University of California at Davis, Sacramento, California, 95817, USA; 3 Department of Cardiothoracic surgery, University of Texas Health Sciences Center in San Antonio, Texas, 78229-3901, USA; 4 Saint George's School of Medicine, Grenada, West Indies and Department of Internal Medicine, San Joaquin County Hospital, 500 West hospital road, French Camp, California, 95231, USA; 5 Department of Gastroenterology, San Joaquin County Hospital, 500 West hospital road, French Camp, California, 95231, USA; 6 Department of Surgery, San Joaquin County Hospital, 500 West hospital road, French Camp, California, 95231, USA

## Abstract

**Background:**

Spontaneous rupture of the esophagus (Boerhaave's syndrome) is a rare, well-defined clinical syndrome caused by a longitudinal perforation of the esophagus. It is a life-threatening condition that necessitates rapid diagnosis and treatment. Patients typically present acutely with a history of vomiting followed by chest or abdominal pain. However, the diagnosis may be difficult or missed when patients present with chronic symptoms that mimic other conditions.

**Case Presentation:**

In this report, we present a unique case of Boerhaave's syndrome in a 53-year-old male patient. In contrast to the more common acute presentation, our patient developed non-specific symptoms in association with an intrathoracic cyst. In this report, we will also review the usual presenting signs, symptoms, and treatment of Boerhaave's syndrome.

**Conclusion:**

Our emphasis in this paper will be on the importance of recognizing and diagnosing Boerhaave's syndrome in an acute as well as a chronic state.

## Background

Boerhaave's Syndrome is a condition of spontaneous, longitudinal esophageal tear due to the elevated intraesophageal pressure that that classically follows repeated episodes of vomiting. Since the distal third of the esophagus is inherently weaker than the rest of the esophagus, [1,2] rupture is seen most often in this segment. Of the different types of spontaneous rupture of the esophageal wall, the tear in Boerhaave's syndrome is full thickness, whereas a Mallory-Weiss tear involves only the mucosa [3,4].

Furthermore, presentations of esophageal perforation can be distinguished as acute, subacute, and chronic. Acute perforation presents with symptoms within twenty-four hours after rupture. In a subacute rupture, symptoms develop between twenty four hours to two weeks following perforation. With chronic perforation, the onset of symptoms is more insidious, often delaying presentation and diagnosis for weeks to months after rupture [5,6].

A history of forceful emesis, subxiphoid chest pain, and subcutaneous emphysema (termed the Mackler Triad) is a common triad of symptoms that should suggest acute esophageal rupture [3,4]. However, atypical presentations in which esophageal rupture mimics pneumonia, myocardial infarction, or aortic aneurysm are sometimes seen as well. Chest films may reveal pneumomediastinum, unilateral effusion, pneumothorax, hydropneumothorax, subcutaneous emphysema, or mediastinal widening. Additionally, x-rays may show radiolucent streaks of air which dissect through fascial planes behind the heart in the shape of the letter "V", the so-called "V-sign" of Naclerio [7,8]. Other imaging techniques to consider in diagnosing esophageal rupture are barium esophagram and CT chest [9]. In both techniques, there will be evidence of extravasation of food particles or bile from the esophageal lumen into the pleural space or mediastinum [5]. Finally, endoscopy can identify the location of the esophageal defect and confirm the existence of extra-luminal disease or to rule out the diagnosis altogether.

Treatment of spontaneous esophageal rupture can be either non-operative or operative. Nonoperative treatment is best for patients with a contained perforation and the absence of clinical mediastinitis [10,11]. Such therapy usually includes targeted drainage, intravenous antibiotics, nasogastric decompression, and enteral nutrition. Patients who are either unstable, have clinically significant mediastinitis, or a non-contained rupture, generally require surgery. Guidelines for operative intervention in Boerhaave's syndrome, are based upon perforation size and amount of adjacent contamination [12]. When the rupture is larger than 1 cm with considerable mediastinal contamination, the recommended treatment is T-tube controlled esophagocutaneous fistula, which allows esophageal drainage and promotes healing of the surrounding structures. With amore severe case of rupture, thoracotomy with direct repair may be necessary. Less invasive techniques such as video assisted thorascopic surgery (VATS) [13], endoscopic clipping [14], and placement of endoluminal stents [15] can serve as alternatives to thoracotomy.

## Case Presentation

A 53-year-old Caucasian male with a history of heavy nonsteroidal anti-inflammatory drug (NSAID) usage and chronic alcohol abuse presented to the emergency room with a four-month history of dysphagia, 45-pound weight loss, coffee-ground vomitus and upper abdominal pain. Physical examination was normal, except for nonspecific epigastric tenderness, as were routine laboratory studies including CBC, chemistries and liver function studies. Although chest X-rays showed only mild perihilar consolidation, esophagogastroduodenoscopy (EGD) confirmed the presence of a large, intrathoracic, cystic structure, partially filled with necrotic debris, arising from a large entry orifice just above the gastroesophegal junction. Computed tomography (CT) of the chest demonstrated bilateral pleural effusions, pneumomediastinum, and a 6.7 cm diameter, fluid-filled diverticulum extending from the esophagus into the posteromedial left hemithorax, Fluoroscopy, performed during injection of barium through a nasogastric tube positioned in the distal esophagus, showed contrast extravasation from the distal esophagus into this cavity in the left chest.

The patient was treated with targeted drainage, intravenous antibiotics, nasogastric decompression, and enteral nutrition. However, he became septic two days after the EGD, and required emergency thoracotomy with distal esophagectomy following several days later. After ashort term of ventilator support and parenteral nutrition, he was weaned off of fboth, and was discharged with a PEG tube for enteral feedings, and is currently being evaluated for esophagostomy reversal.

## Conclusion

While a spontaneous esophageal rupture usually presents in an acute setting, it can also present in a subacute or chronic manner as well. Similarly, though patients usually have symptoms of vomiting, chest pain, tachycardia, and tachypnea, a patient with Boerhaave's Syndrome may also present with non-specific complaints and no major physical findings. Due to this diverse range of presentations, the diagnosis of Boerhaave's Syndrome can be difficult, and effective treatments are often delayed. Thus, the diagnosis of Boerhaave Syndrome must be considered in any clinical setting in which a patient presents with a highly suspicious history with or without specific complaints.

## Consent

Written informed consent was obtained from the patient for publication of this case report and accompanying images. A copy of the written consent is available for review by the Editor-in-Chief of this journal.

## Competing interests

The authors declare that they have no competing interests.

## Authors' contributions

UFM was involved in writing, reviewing and editing the manuscript. He was the corresponding author who drafted the initial article. He also finalized the manuscript. RY was involved in writing, editing and reviewing the manuscript. HDP reviewed and edited the manuscript. AMC: was involved in initial writing and editing of the manuscript. BS: was involved in writing, gathering and organizing pictures and final editing of the manuscript. RL was the chief gastroenterologist involved with the case. He also reviewed, edited and finalized the manuscript. AM was the chief of surgery and attending involved with the case. He also reviewed and finalized the manuscript.

**All authors read and approved the final manuscript**.

## Pre-publication history

The pre-publication history for this paper can be accessed here:

http://www.biomedcentral.com/1471-230X/10/29/prepub
